# Posttranslational Modifications in Ferroptosis

**DOI:** 10.1155/2020/8832043

**Published:** 2020-11-26

**Authors:** Xiang Wei, Xin Yi, Xue-Hai Zhu, Ding-Sheng Jiang

**Affiliations:** ^1^Division of Cardiothoracic and Vascular Surgery, Tongji Hospital, Tongji Medical College, Huazhong University of Science and Technology, Wuhan 430030, China; ^2^Key Laboratory of Organ Transplantation, Ministry of Education, Wuhan 430030, China; ^3^NHC Key Laboratory of Organ Transplantation, Wuhan 430030, China; ^4^Key Laboratory of Organ Transplantation, Chinese Academy of Medical Sciences, Wuhan 430030, China; ^5^Department of Cardiology, Renmin Hospital of Wuhan University, Wuhan 430060, China

## Abstract

Ferroptosis was first coined in 2012 to describe the form of regulated cell death (RCD) characterized by iron-dependent lipid peroxidation. To date, ferroptosis has been implicated in many diseases, such as carcinogenesis, degenerative diseases (e.g., Huntington's, Alzheimer's, and Parkinson's diseases), ischemia-reperfusion injury, and cardiovascular diseases. Previous studies have identified numerous targets involved in ferroptosis; for example, acyl-CoA synthetase long-chain family member 4 (ACSL4) and p53 induce while glutathione peroxidase 4 (GPX4) and apoptosis-inducing factor mitochondria-associated 2 (AIFM2, also known as FSP1) inhibit ferroptosis. At least three major pathways (the glutathione-GPX4, FSP1-coenzyme Q_10_ (CoQ_10_), and GTP cyclohydrolase-1- (GCH1-) tetrahydrobiopterin (BH4) pathways) have been identified to participate in ferroptosis regulation. Recent advances have also highlighted the crucial roles of posttranslational modifications (PTMs) of proteins in ferroptosis. Here, we summarize the recently discovered knowledge regarding the mechanisms underlying ferroptosis, particularly the roles of PTMs in ferroptosis regulation.

## 1. Introduction

Cell death is critical for the development of multicellular organisms and participates in degenerative diseases. Over the past decades, more than ten types of cell death have been discovered and defined according to their different morphological characteristics, biomarkers, or regulatory mechanisms, and these include apoptosis, necroptosis, pyroptosis, and autophagy-dependent cell death [1–4]. Ferroptosis was first coined in 2012 to describe the form of regulated cell death (RCD) driven by the iron-dependent accumulation of lipid hydroperoxides [5]. Numerous metabolic pathways involving the metabolism of polyunsaturated fatty acids (PUFAs), iron, and amino acids and the biosynthesis of glutathione, nicotinamide adenine dinucleotide phosphate (NADPH), coenzyme Q10 (CoQ_10_), and phospholipids are tightly linked to the sensitivity of cells to ferroptosis [5–7]. Emerging evidence suggests that ferroptosis is involved in both normal physiological contexts and pathological diseases in mammals, including degenerative diseases (e.g., Parkinson's, Huntington's, and Alzheimer's diseases), ischemia-reperfusion injury, and carcinogenesis [6, 7]. Recent studies have shed light on the mechanisms how posttranslational modifications (PTMs) of protein regulates ferroptosis. In this review, we summarize these recent advances in the understanding of how protein phosphorylation, ubiquitination, acetylation, and methylation affect iron metabolism, lipid oxidation, and ferroptosis.

## 2. Major Pathways Regulating Ferroptosis

### 2.1. Glutathione-Glutathione Peroxidase 4 (GPX4) Pathway in Ferroptosis

**Figure 1:**
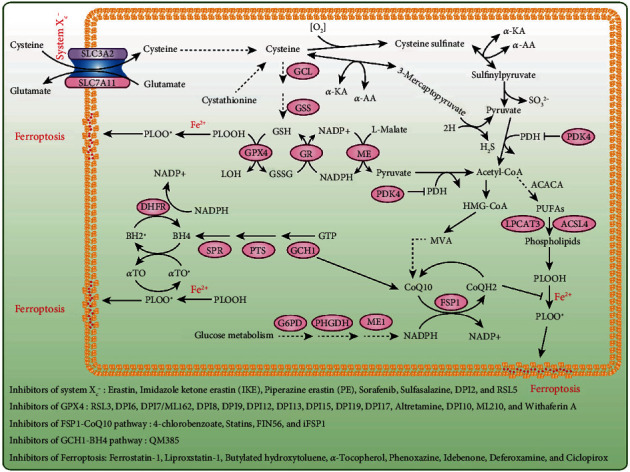
The major pathways regulate ferroptosis. Three major pathways have been identified in ferroptosis, including the glutathione-GPX4, NADPH-FSP1-CoQ_10_, and GCH1-BH4 pathways. Chemical probes regulating these pathways and ferroptosis are shown at the bottom of this figure. Abbreviations: SLC7A11: solute carrier family 7 member 11; GCL: glutamate cysteine ligase; GSS: glutathione synthetase; GPX4: glutathione peroxidase 4; GR: glutathione reductase; ME: malic enzyme; PDK4: pyruvate dehydrogenase kinase 4; LPCAT3: lysophosphatidylcholine acyltransferase 3; ACSL4: acyl-CoA synthetase long-chain family member 4; FSP1: ferroptosis suppressor protein 1; G6PD: glucose-6-phosphate dehydrogenase; PHGDH: phosphoglycerate dehydrogenase; ME1: malic enzyme 1; GCH1: GTP cyclohydrolase 1; PTS: 6-pyruvoyltetrahydropterin synthase; SPR: sepiapterin reductase; DHFR: dihydrofolate reductase.

The selenoprotein GPX4 was the first identified central inhibitor of ferroptosis [8] (Figure 1). Glutathione- (GSH-) dependent GPX4 can reduce lipid peroxides (PL-PUFA-OOH) which serves as the major trigger of ferroptosis [9]. It is well known that the constitutive deletion of *GPX4* or the expression of inactive *GPX4* causes embryonic death [10, 11]. However, Ingold et al. found that the selenolate-based catalysis of GPX4 is not essential for normal embryogenesis [12]. Thus, conditional *GPX4* knockout mice were generated to analyze the cell death mechanisms induced by *GPX4* inhibition [13–16]. Unexpectedly, lipid peroxidation was observed in all these knockout models, which indicated that the accumulation of lipid peroxides is an important cause of embryonic death. More importantly, *GPX4* knockout-induced cell death is largely reversed by the ferroptosis inhibitor liproxstatin-1 in a preclinical model of ischemia/reperfusion-induced hepatic damage [17]. Yang et al. revealed that GPX4 overexpression and knockdown regulate the cell death of 12 ferroptosis inducers (e.g., erastin and RSL3) but not of 11 compounds with other lethal mechanisms [8]. Furthermore, after screening 177 cancer cell lines, these researchers found that renal cell carcinomas and diffuse large B cell lymphomas are more sensitive to GPX4-regulated ferroptosis [8]. Because GPX4 is a selenoprotein, pharmacological selenium (Se) delivered into the brain augments GPX4 expression, and this effect inhibits ferroptotic death as well as cell death induced by excitotoxicity or ER stress to protect neurons and improve behavior in a hemorrhagic stroke model [18]. Ingold et al. also demonstrated that selenocysteine-containing GPX4 is indispensable for the survival of a specific type of interneuron and thereby protects against fatal epileptic seizures [12]. These studies indicate that GPX4 is a vital regulator of ferroptosis. Therefore, GPX4 is a potential therapeutic target for degenerative diseases (activation of GPX4) and tumors (inactivation of GPX4).

The activity of GPX4 is not only directly inhibited by RSL3 but also inhibited by erastin or cystine deprivation through GSH depletion [19, 20]. Because system X_c_- is the cysteine-glutamate antiporter, its activity is closely associated with the amount of GSH in the cell, and thus, this complex plays an important regulatory role in ferroptosis [5, 21]. Although the germline deletion of solute carrier family 7 member 11 (*Slc7a11*) is well tolerated in unstressed mice [22], *Slc7a11* deficiency induces tumor-selective ferroptosis and inhibits pancreatic ductal adenocarcinoma (PDAC) growth [23]. PDAC cells use cysteine to synthesize glutathione and coenzyme A to suppress ferroptosis, and the depletion of cysteine and cystine by cyst(e)inase induces ferroptosis in PDAC [23]. Jiang et al. found that p53 inhibits cystine uptake and sensitizes cells to ferroptosis by repressing SLC7A11 expression [24]. Furthermore, SLC7A11 directly interacts with arachidonate (12S)-lipoxygenase (ALOX12) to inhibit its enzymatic activity [25]. The loss of one *Alox12* allele abrogates PUFA peroxidation and p53-mediated ferroptosis to accelerate tumorigenesis [25]. Moreover, Wang et al. found that immunotherapy-activated CD8^+^ T cells release interferon gamma (IFN*γ*), which upregulates interferon regulatory factor 1 (IRF1) and downregulates SLC7A11 and SLC3A2 in cancer cells to promote tumor cell lipid peroxidation and sensitize tumors to ferroptosis [26].

### 2.2. NADPH-FSP1-CoQ_10_ Pathway in Ferroptosis

GPX4 is considered the primary regulator inhibiting ferroptosis, but in some cell types or cell lines, the inhibition of GPX4 fails to induce ferroptosis, which indicates the existence of alternative mechanisms [7, 27, 28]. Therefore, to uncover the potential factors that inhibit ferroptosis via an independent GPX4 pathway, Doll et al. generated a cDNA expression library derived from a ferroptosis-resistant cell line to screen genes complementing GPX4 loss [27, 29, 30]. These researchers revealed that apoptosis-inducing factor mitochondria-associated 2 (AIFM2, also known as FSP1) overexpression can largely abrogate GPX4 inhibition-induced ferroptosis [27, 31]. Similarly, using a synthetic lethal CRISPR/Cas9 screen employing an apoptosis and cancer single-guide RNA (sgRNA) sublibrary of cells treated with RSL3, Bersuker et al. verified that FSP1 acts as a novel ferroptosis suppressor that induces its effect via a GPX4-independent mechanism [28]. However, the overexpression of AIFM1, which is homologous to FSP1, exhibits almost no activity in suppressing ferroptosis [27, 31, 32]. Because the N-terminus of FSP1 contains a canonical myristoylation motif and myristoylation is a lipid modification that promotes target protein binding to cell membranes [27, 33, 34], both research groups demonstrated that myristoylation at the N-terminus of the FSP1 protein facilitates the localization of FSP1 to the plasma membrane, which is critical for its ferroptosis-inhibitory activity [27, 28]. A previous study showed that FSP1 functions as an nicotinamide-adenine dinucleotide phosphate- (NADP-) dependent coenzyme Q (CoQ) oxidoreductase in vitro [35]. CoQ_10_ is a mobile lipophilic electron carrier that endogenously synthesizes lipid-soluble antioxidants and acts as a lipophilic free radical-trapping agents (RTAs) in the plasma membrane [36, 37]. FSP1 overexpression fails to suppress ferroptosis in both 4-hydroxybenzoate polyprenyltransferase (CoQ_2_) knockout cells and in cells treated with the CoQ_2_ inhibitor 4-chlorobenzoic acid (4-CBA) [27, 28]. CoQ_2_ is the enzyme that catalyzes the first step in CoQ_10_ biosynthesis, and idebenone, a soluble analog of CoQ_10_, is sufficient for suppressing ferroptosis and lipid peroxidation [27, 28]. These two latest studies clearly suggest that FSP1 acts parallel to GPX4 to inhibit ferroptosis by regulating the nonmitochondrial CoQ_10_ antioxidant system (Figure 1). The combined pharmacological inhibition of FSP1 and GPX4 might be an effective strategy for sensitizing cancer cells, particularly cancer cells that are not sensitive to a GPX4 inhibitor alone, to ferroptosis-inducing chemotherapeutics.

### 2.3. GCH1-BH4 Pathway in Ferroptosis

Tetrahydrobiopterin (BH4) is a redox-active cofactor involved in the production of nitric oxide, neurotransmitters, and aromatic amino acids [38, 39]. The GTP cyclohydrolase-1- (GCH1-) 6-pyruvoyltetrahydropterin synthase- (PTS-) sepiapterin reductase (SPR) pathway catalyzes GTP to BH4, and GCH1 is a rate-limiting enzyme in the synthesis of BH4 [40–42]. BH4 exhibits antioxidant properties in vitro [41]. However, the role of the GCH1-BH4 pathway in ferroptosis has not been elucidated until recently, when two independent research teams identified GCH1-BH4 as a novel pathway that regulates ferroptosis through the use of metabolism-focused CRISPR-Cas9 genetic screens and a genome-wide dCas9-based activation screen (CRISPRa) [39, 42]. Kraft et al. found that the overexpression of GCH1, MS4A15, and OLFR367-ps not only abolishes lipid peroxidation but also yields almost complete protection against ferroptosis [39]. GCH1 overexpression exhibits robust protection against RSL3- and IKE-induced ferroptosis and genetic ablation of *GPX4*-induced ferroptosis but does not protect cells against inducers of apoptosis and is only marginally effective against necroptosis, which indicates that GCH1 selectively counters ferroptotic cell death [39]. Furthermore, the protective role of GCH1 on ferroptosis is independent of the known ferroptosis pathway-related proteins or the glutathione system [39]. Soula et al. demonstrated that the deletion of GCH1 or SPR, as well as the inhibition of SPR with QM385, sensitizes cells to RSL3 but not erastin treatment in Jurkat cells [42]. The supplementation of ferroptosis inducers-treated cells with BH2 or BH4 is sufficient to rescue cells from ferroptosis [39, 42]. Although BH4 serves as a cofactor for several biosynthetic enzymes, both research teams found that this function of BH4 does not play a relevant role in its protective effect against ferroptosis [39, 42]. Intriguingly, the accumulation of coenzyme A, NADP, and oxidized GSH (GSSG) in cells with BH4 loss and the elevation of reduced CoQ_10_ in cells with GCH1 overexpression have been detected [39, 42]. Thus, these results indicate that the GCH1-BH4 pathway acts as an endogenous antioxidant pathway to inhibit ferroptosis through a mechanism independent of the GPX4/glutathione system (Figure 1).

## 3. Inducers and Inhibitors of Ferroptosis

### 3.1. Inducers of Ferroptotic Death

#### 3.1.1. Inhibitors of System X_c_-

In 2003, Dolma et al. identified a small molecule (erastin) from a diverse chemical library that selectively kills engineered tumor cells through a nonapoptotic mechanism [43]. Erastin was the first discovered inducer of ferroptosis, and this molecules induces ferroptosis by inhibiting the activity of the cysteine-glutamate antiporter, which is a complex composed of SLC7A11 and SLC3A2 and is also known as system X_c_- [5, 21]. The inhibition of cystine uptake by erastin leads to depletion of the intracellular reduced and oxidized forms of glutathione (GSH and GSSG) and subsequent accumulation of peroxidized phospholipids, which triggers ferroptotic cell death [8, 21]. Imidazole ketone erastin (IKE) and piperazine erastin (PE) are derivatives of erastin with better pharmacological properties for inducing ferroptosis, and both derivatives are suitable for *in vitro* and *in vivo* studies [8, 44, 45]. Some other inhibitors, such as sorafenib and sulfasalazine, can also inhibit system X_c_- but with lower potency and less selectivity than erastin and its derivatives [21, 46]. The compounds DPI2 and RSL5 have the potential to inhibit system X_c_-, but further validation is needed [8, 47] (Figure 1).

#### 3.1.2. Inhibitors of Glutathione Peroxidase 4 (GPX4)

GPX4 is a glutathione peroxidase that catalyzes the reduction of hydrogen peroxide, organic hydroperoxides, and lipid hydroperoxides and thereby protects cells against oxidative damage and ferroptosis [48]. RSL3 is a compound that covalently inhibits GPX4 in an irreversible manner [8, 49]. Although RLS3 is widely used to induce the ferroptosis of cultured cells *in vitro*, its poor solubility and unfavorable absorption, distribution, metabolism, and excretion properties hinder the *in vivo* application of RSL3 [19]. In addition to RSL3, several other chloroacetamide-containing inhibitors of GPX4 have been identified, and these include DPI6, DPI7/ML162, DPI8, DPI9, DPI12, DPI13, DPI15 and DPI19 [8]. Three additional structural classes of GPX4 inhibitors have been reported: chloromethyltriazines (e.g., DPI17 and altretamine), nitroisoxazoles (e.g., DPI10 and ML210), and steroidal lactones (e.g., withaferin A) [19] (Figure 1).

#### 3.1.3. Inhibitors Regulating the Lipophilic Antioxidant Ubiquinol/Coenzyme Q_10_ (CoQ_10_) Pathway

CoQ_10_, a key compound of the mevalonate pathway, is a fat-soluble compound needed for energy generation in the mitochondrial electron transport chain and in membranes of lysosomes [50]. Shimada et al. reported that the inhibition of CoQ_10_ can sensitize cells to ferroptosis in some contexts [51]. For example, 4-chlorobenzoate can inhibit CoQ_10_ production in cells to activate ferroptosis with low potency [28]. Statins are a class of lipid-lowering drugs that inhibit the enzyme HMG-CoA reductase, which is a rate-limiting enzyme that catalyzes the conversion of HMG-CoA to mevalonic acid in the mevalonate pathway [52]. In addition to statins, these researchers also reported that FIN56 presumably depletes CoQ_10_ by regulating the mevalonate pathway to facilitate ferroptosis [51]. Furthermore, two recent studies demonstrated that ferroptosis suppressor protein 1 (FSP1), which was previously called AIFM2, functions as a CoQ_10_ reductase to suppress ferroptosis, and iFSP1, a selective inhibitor of FSP1, induces ferroptosis through a GPX4-independent mechanism [27, 28] (Figure 1).

#### 3.1.4. Other Inducers of Ferroptosis

Cystine deprivation or cysteine depletion is an effective means for inducing ferroptosis [20, 23]. Cyst(e)inase, an engineered enzyme that degrades both cystine and cysteine in the circulation efficiently induces lipid oxidation and results in ferroptosis [23, 26]. FINO2 oxidizes iron to inactivate GPX4 enzymatic activity but does not directly target GPX4, deplete CoQ_10_, or inhibit system X_c_- [53, 54]. In addition, several research groups have indicated that radiation induces ferroptosis [55–58], and the results indicate that other ferroptosis inducers could act as radiosensitizers, whereas inhibitors of ferroptosis might offset the effects of radiation in cancer.

### 3.2. Inhibitors of Ferroptotic Death

Lipid peroxidation is one of the major features of ferroptosis, and chemical probes that interrupt this process could serve as ferroptosis inhibitors. To date, several small-molecule RTAs, including ferrostatin-1 (Fer-1), liproxstatin-1, phenoxazine, and *α*-tocopherol, have been found to suppress ferroptosis [5, 17, 59]. Although ferrostatin-1 is widely used in *in vitro* experiments, it should be used with caution when conducting *in vivo* studies due to its low metabolic stability [60]. However, liproxstatin-1 performs better in this regard [17]. Moreover, both ferrostatin-1 and liproxstatin-1 exhibit good specificity for inhibiting ferroptosis with no obvious off-target effects [19], whereas *α*-tocopherol has moderate potencies as a ferroptosis suppressor [59]. Because CoQ_10_ suppresses lipid peroxidation and ferroptosis in some contexts [27, 28, 51], the CoQ_10_ analog idebenone has been used to mimic the antiferroptosis effect of CoQ_10_ [19, 51]. In addition, Cu(II)atsm and deuterated PUFAs have been reported to suppress lipid peroxidation and ferroptosis [61–63]. Iron chelators, such as deferoxamine and ciclopirox, have also been used as ferroptosis suppressors [5] (Figure 1).

## 4. Posttranslational Modifications (PTMs) in Ferroptosis

PTMs include phosphorylation, acetylation, ubiquitination, and methylation, SUMOylation, and most PTMs are reversible [64]. These PTMs regulate the activity and stability of target proteins, protein interactions, and intracellular distribution [65]. PTMs not only make the functions of proteins more diverse but also act as a switch to enable cells or organisms to rapidly and strictly respond to stress [64]. The role of PTMs of proteins in ferroptosis has gradually been highlighted in recent years [66].

### 4.1. Phosphorylation in Ferroptosis

**Figure 2:**
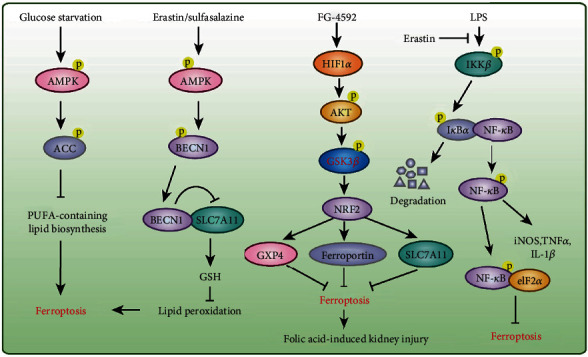
Protein phosphorylation in ferroptosis. Phosphorylation of AMPK, AKT, and NF-*κ*B were involved in the regulation of ferroptosis under specific stimulations.

Phosphorylation is the most common type of PTM involved in the regulation of protein stability and enzyme activity, and these PTMs usually occur on serine, tyrosine, and threonine residues of the targeted protein [64, 67]. Energy stress induces cell death, and this effect is associated with reactive oxygen species (ROS) induction [68]. Because lipid peroxidation is the main feature of ferroptosis, the relationship between energy stress and ferroptosis seems predictable, that is, energy stress facilitates ferroptosis. However, unexpected results are always observed. Specifically, Lee et al. demonstrated that glucose starvation unexpectedly suppresses erastin-induced ferroptosis in mouse embryonic fibroblasts (MEFs), and this effect has also been observed with cystine depletion-, RSL3-, and GPX4 deletion-induced ferroptosis [69]. AMP-activated protein kinase (AMPK) is a critical sensor of the cellular energy status, and glucose starvation results in the phosphorylation of AMPK and its activation [70]. The inhibitory effect of glucose starvation on ferroptosis was largely abolished by *Ampkα1/α2* double knockout (DKO) both *in vitro* in MEFs and *in vivo* in a mouse model of renal ischemia-reperfusion (I/R) injury [69]. Mechanistically, AMPK accelerates the phosphorylation of acetyl-CoA carboxylase (ACC) and suppresses PUFA-containing lipid biosynthesis to mediate the inhibitory effect of glucose starvation on ferroptosis [69]. Moreover, cancer cells with high basal AMPK activation are resistant to ferroptosis and AMPK inactivation sensitizes these cells to ferroptosis [69]. These results indicate that AMPK is a negative regulator of ferroptosis (Figure 2).

However, Song et al. demonstrated that the ferroptosis inducers erastin and sulfasalazine activate AMPK, which phosphorylates BECN1 at Ser90/93/96 and thereby facilitates BECN1-SLC7A11 complex formation [71] (Figure 2). The interaction between BECN1 and SLC7A11 directly blocks system X_c_- to promote ferroptosis. Furthermore, the knockdown of *BECN1* represses system X_c_- inhibitor (e.g., erastin and sulfasalazine)-induced ferroptosis but not that induced by RSL3, FIN56, or buthionine sulfoximine; conversely, the overexpression of *BECN1* or the administration of the BECN1 activator peptide Tat-Beclin 1 promotes cancer cell ferroptosis *in vitro* and *in vivo* [71]. Although the autophagy machinery (e.g., ATG5, ATG7, ATG4B, ATG13, and NCOA4) is involved in ferroptotic cell death and BECN1 is a key player in autophagy [72, 73], the knockdown of *BECN1* does not affect the formation of lipidated microtubule-associated protein 1 light chain 3 (MAP1LC3B) and MAP1LC3B-positive puncta in ferroptosis [71, 74]. These findings suggest that BECN1 cooperates with different partners to play distinct roles in autophagy (BECN1-PtdIns3K complex) and ferroptosis (BECN1-SLC7A11 complex) in the presence of specific stimuli. In addition, the benzopyran derivative 2-imino-6-methoxy-2H-chromene-3-carbothioamide (IMCA) reportedly accelerate the ferroptosis of colorectal cancer cells by activating AMPK and inhibiting the SLC7A11 and mTOR-p70S6K signaling pathway [75].

Therefore, the aforementioned studies did not reach a unified conclusion regarding the effect of AMPK on ferroptosis. Because metformin is an AMPK agonist that not only lowers glycemia but also has great potential to relieve tumors [76, 77], whether ferroptosis is involved in the antitumor effect remains unknown. Because the role of AMPK in ferroptosis remains controversial [69, 71], clarifying the effect of metformin on ferroptosis of tumor cells would be of great significance.

The Toll-like receptor 4- (TLR4-) nuclear factor kappa-light-chain-enhancer of activated B cells (NF-*κ*B) signaling pathway activates the expression of several proinflammatory cytokine genes that play pivotal roles in inflammatory disorders, such as sepsis, ulcerative colitis, myocardial infarction, and I/R injury [78–80]. The pretreatment of bone marrow-derived macrophages (BMDMs) with the ferroptosis inducer erastin significantly attenuates the expression of proinflammatory cytokines (e.g., inducible nitric oxide synthase, tumor necrosis factor- (TNF-) *α*, and interleukin- (IL-) 1*β*) induced by lipopolysaccharide (LPS) treatment, and these effects are mediated by inhibition of the phosphorylation of I*κ*B kinase *β* (IKK*β*) and the phosphorylation and degradation of I*κ*B*α* and NF-*κ*B and thereby lead to the suppression of sepsis development [80]. Ferroptosis is detected in intestinal epithelial cells from patients with ulcerative colitis and mice with colitis, and Fer-1 alleviates experimental colitis [79]. Furthermore, phosphorylated-NF-*κ*B directly binds to eukaryotic initiation factor 2*α* (eIF2*α*) to suppress the ER-stress-mediated ferroptosis of intestinal epithelial cells to alleviate ulcerative colitis [79] (Figure 2). A recent study demonstrated that the inhibition of ferroptosis by Fer-1 or iron chelation mitigates heart failure induced by both acute and chronic I/R in mice [81]. Li et al. also found that Fer-1 reduces the infarct size and left ventricular remodeling and improves cardiac function in an I/R mouse model [82]. Moreover, Fer-1 reduces cardiomyocyte cell death and blocks the adhesion of neutrophils to coronary vascular endothelial cells by regulating the TLR4/TIR domain-containing adapter molecule 1 (TRIF)/type I interferon (IFN) signaling pathway following heart transplantation [82].

In addition, folic acid-induced kidney injury is alleviated by pretreatment with FG-4592, an inhibitor of prolyl hydroxylase of hypoxia-inducible factor (HIF), which increases the phosphorylation of protein kinase B (Akt) and glycogen synthase kinase 3*β* (GSK-3*β*) and activates NFE2-related factor 2 (Nrf2) to inhibit ferroptosis in mice [83] (Figure 2). Protein kinase C-mediated heat shock protein beta-1 (HSPB1) phosphorylation confers protection against erastin-induced ferroptosis by reducing lipid peroxidation [84].

### 4.2. Ubiquitination in Ferroptosis

**Figure 3:**
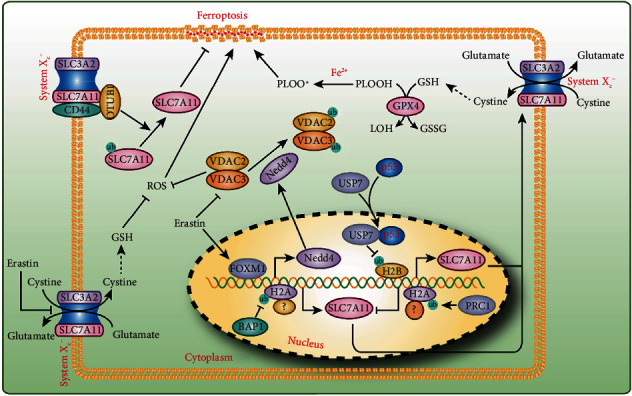
Protein ubiquitination in ferroptosis. Both SLC7A11 expression and its stability were regulated by ubiquitination in ferroptosis. Ubiquitination of H2A and H2B was critical for SLC7A11 expression, while OTUB1 deubiquitinating SLC7A11 increases its stability. Erastin not only inhibits the activity of system Xc^−^ but also regulates the ubiquitination of VDAC2/3 via Nedd4 to promote ferroptosis.

Ubiquitination is a well-known PTM and involves the covalent addition of ubiquitin to the lysine residues of target protein to regulate its degradation and turnover [85, 86]. Recent findings demonstrate that the ubiquitination of proteins plays a critical regulatory role in ferroptosis [87]. Cancer cells challenged with palladium pyrithione complex (PdPT), a pan-deubiquitinase (pan-DUB) inhibitor, undergo apoptosis and ferroptosis with caspase activation and GPX4 protein degradation [87]. However, the mechanism through which GPX4 is ubiquitinated and the sites of ubiquitination require more in-depth research. BRCA1-associated protein 1 (BAP1) is a DUB enzyme that reduces histone 2A ubiquitination (H2Aub) [88]. In cancer cells, BAP1 removes monoubiquitin from ubiquitinated H2A at lysine 119 (H2Aub) on the SLC7A11 promoter to suppress its expression in cells treated with erastin but not RSL3, and this effect does not require the transcription factors NRF2 and activating transcription factor 4 (ATF4) [89, 90]. Decreased SLC7A11 inhibits cystine uptake, which leads to elevated lipid peroxidation and ferroptosis (Figure 3). Moreover, cancer-associated BAP1 mutants lose its inhibition of SLC7A11 and repress ferroptosis [89]. Intriguingly, polycomb repressive complex 1 (PRC1), a ubiquitin ligase that monoubiquitinates H2A at lysine 119, also suppresses SLC7A11 expression [90] (Figure 3). Because the expression levels of components of PRC1 and PRC2 (e.g., BMI1, RNF2, and H3K27me3) are not affected by BAP1 [90], an in-depth study of why both BAP1 and PRC1 can inhibit the expression of SLC7A11 would yield interesting findings.

In addition to H2Aub, the monoubiquitination of histone H2B on lysine 120 (H2Bub) has also been shown to be involved in the regulation of ferroptosis [91]. Wang et al. found lower levels of H2Bub in erastin-induced cells compared with control cells. Their further research showed that the tumor suppressor p53 facilitates the nuclear translocation of the deubiquitinase ubiquitin-specific peptidase 7 (USP7) to negatively regulate the H2Bub levels in a transcriptional-independent manner, and this effect decreases SLC7A11 expression during erastin treatment [91] (Figure 3). Instead, OTU deubiquitinase ubiquitin aldehyde binding 1 (OTUB1) directly interacts with SLC7A11 and stabilizes SLC7A11 by deubiquitinating SLC7A11, and this interaction is tightly regulated by the cancer stem cell marker CD44 [92] (Figure 3). Erastin induces ferroptosis partially by binding to and inhibiting the voltage-dependent anion channels VDAC2 and VDCA3 [19], and these effects lead to the degradation of the channels [93]. Yang et al. demonstrated that erastin induces Nedd4 expression by inducing FOXM1, which increases the K48-linked ubiquitination of VDAC2/3 and then its degradation [93] (Figure 3). Androgen receptor (AR), a steroid hormone receptor, is a well-recognized biomarker for predicting prognosis in prostate cancer [94]. ALZ003, a curcumin analog, extends the survival period of transplanted mice by promoting glioblastoma cell ferroptosis, and this effect is mediated by F-box and the leucine-rich repeat protein 2- (FBXL2-) dependent ubiquitination of AR and its degradation [94].

### 4.3. Acetylation in Ferroptosis

**Figure 4:**
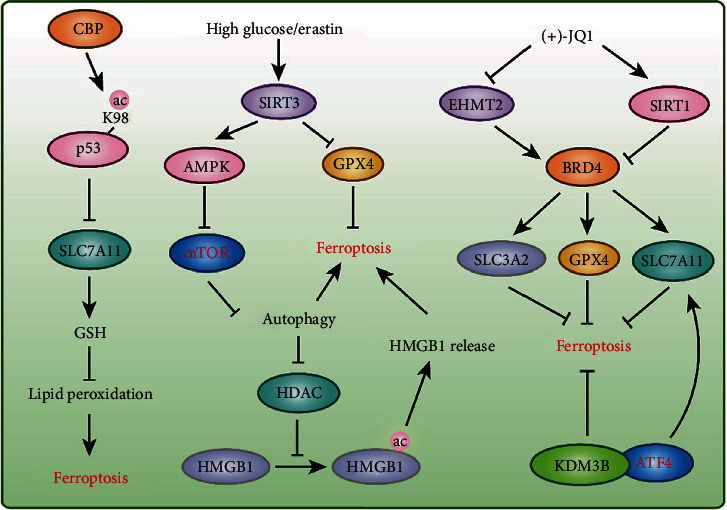
Protein acetylation and methylation in ferroptosis. The acetylation of p53 and HMGB1 participate in ferroptosis regulation. Deacetylases SIRT3, SIRT1, and HDAC were reported to have a function in ferroptosis. Lysine methyltransferase EHMT2 and lysine demethylase KDM3B were involved in ferroptosis via regulating SLC7A11, GPX4, and SLC3A2 expressions, respectively.

Acetylation usually occurs in the lysine residues of target proteins, and this reaction is catalyzed by acetyltransferases and reversed by deacetylases [95]. Acetyltransferases transfer acetyl groups from acetyl coenzyme A (acetyl CoA) to lysine residues, leading to the neutralization of the charge on lysine residues [96]. Acetylation can alter the 3D structure of a protein to affect its ability to bind other proteins or DNA and also regulate the subcellular localization, activity and stability of a protein [96]. The aberrant acetylation of proteins (including histone and nonhistone proteins) is closely related to tumorigenesis [97]. A recent study found that acetylation is involved in the regulation of ferroptosis. Because ferroptosis inducers can induce cancer cell death, these agents have the potential to be used for chemotherapy; however, the toxicity of these agents is a major concern. Zille et al. found that class I histone deacetylase (HDAC) inhibitors selectively protect neurons but augment ferroptosis in cancer cells, which indicates that class I HDAC inhibitors are ideal drugs that not only exert anticancer effect but also protect normal cells [98]. However, more experiments, including studies with other tumors and *in vivo* studies, should be performed to validate these results. Because p53 is the critical regulator of ferroptosis and acetylation is crucial for the activity of p53 [50, 64, 91], the regulatory effect of acetylated p53 on ferroptosis should be clarified. Jiang et al. demonstrated that p53 inhibits SLC7A11 expression to reduce cystine uptake and sensitize cells to ferroptosis; furthermore, p53^3KR^ (K117R, K161R, and K162R), an acetylation-defective mutant, fully retains the ability to suppress SLC7A11 expression but fails to induce cell cycle arrest, senescence, and apoptosis [24]. Further research has shown that p53^4KR^ (K98R+3KR) completely abolishes its ability to regulate SLC7A11, but p53^K98R^ alone exerts very modest effects on p53-mediated transactivation [99]. Moreover, the anticancer effects of p53 are severely defective in mouse xenograft models with p53^4KR^ expression [99]. These results indicate that acetylation is crucial for p53-induced ferroptosis (Figure 4).

Sirtuin 3 (SIRT3) is a prototypical NAD^+^-dependent mitochondrial protein deacetylase that is involved in ROS production and cell death [100]. High concentrations of glucose and ferroptosis inducers stimulate SIRT3 overexpression, which activates the AMPK-mTOR pathway to enhance autophagy and inhibits GPX4 to induce ferroptosis [101]. In addition, autophagy inhibition attenuates SIRT3-enhanced ferroptosis [101] (Figure 4). High-mobility group box 1 (HMGB1) is a damage-associated molecular pattern (DAMP) molecule released by ferroptotic cells in an autophagy-dependent manner, and autophagy-mediated HDAC inhibition facilitates the acetylation and release of HMGB1 [102] (Figure 4). The histone acetyltransferase KAT2B is also involved in ferroptosis by governing the transcription factor hepatocyte nuclear factor 4 alpha (HNF4A) and hypermethylated in cancer 1 (HIC1) to regulate ferroptosis-related gene expression [103].

### 4.4. Methylation in Ferroptosis

Protein methylation usually occurs on lysine or arginine, and both histones and nonhistone proteins can be methylated [104–106]. A plethora of studies have demonstrated that protein methylation plays an important role in cell survival/death and diseases [104, 107–109], but the research on their roles in ferroptosis is in its infancy. For example, lysine demethylase 3B (KDM3B), a histone H3K9 demethylase, inhibits erastin-induced ferroptosis by cooperating with the transcription factor activating transcription factor 4 (ATF4) to upregulate SLC7A11 expression [110] (Figure 4). Bromodomain-containing 4 (BRD4) knockdown and (+)-JQ1, an inhibitor of BRD4, induce ferroptosis via ferritinophagy in breast cancer cell lines [111]. (+)-JQ1 inhibits euchromatic histone lysine methyltransferase 2 (EHMT2; also known as G9a) expression but promotes SIRT1 expression to suppress BRD4, and these effects negatively regulate GPX4, SLC7A11, and SLC3A2 expressions to regulate ferritinophagy [111] (Figure 4). These results indicate that protein methylation plays a vital role in ferroptosis; however, more in-depth studies are needed to further clarify the role and molecular mechanism of protein methylation in ferroptosis. For example, does the methylation of nonhistone proteins (e.g., p53) participate in the regulation of ferroptosis? How do methylation and other PTMs coordinate with each other to regulate ferroptosis?

## 5. Conclusion and Perspectives

Due to the growing number of laboratories devoted to investigating the mechanisms and functions of ferroptosis, the research on ferroptosis is unprecedentedly prosperous. Recent studies have shown that ferroptosis plays a critical role in many diseases, including tumorigenesis, I/R injury, neurological disorders, and cardiovascular diseases [6, 72, 112–114]. At least three major pathways, namely, the glutathione-GPX4, FSP1-CoQ_10_, and GCH1-BH4 pathways, are involved in ferroptosis regulation [6]. Moreover, a variety of PTMs of proteins (e.g., phosphorylation, ubiquitination, acetylation, and methylation) also play an indispensable regulatory role in ferroptosis. However, many very important and interesting issues have not been elucidated. First, whether ferroptosis plays a role in normal physiological functions or embryonic development is unclear. Second, given that the accumulation of oxidized phospholipids serves as a death signal, the mechanism through which lipid peroxidation leads to ferroptosis is uncovered. Third, due to the lack of specific molecular markers and morphological characteristics, the experimental confirmation of ferroptotic cell death has mainly relied on detecting cellular ROS and the application of ferroptosis inhibitors to reverse cell death, which greatly limits *in vivo* studies and elucidation of the physiological functions of ferroptosis. Fourth, in addition to the phosphorylation, ubiquitination, acetylation, and methylation of proteins, whether other PTMs are involved in regulating ferroptosis remains unclear. Moreover, the mechanism through which these PTMs work together in the ferroptosis process has not been elucidated. Fifth, because ferroptosis can be induced through many different mechanisms, clarifying the specific and shared PTMs that play roles under different conditions that induce ferroptosis would be interesting. Sixth, numerous targets and their PTMs that induce and inhibit ferroptosis have been identified, and whether drugs targeting these targets can be transformed to clinical treatment for patients and their safety and efficacy need further investigation.

## Abbreviations

RCD: Regulated cell death
ACSL4: Acyl-CoA synthetase long-chain family member 4
GPX4: Glutathione peroxidase 4
AIFM2: Apoptosis-inducing factor mitochondria-associated 2
CoQ10: Coenzyme Q10
GCH1: GTP cyclohydrolase-1
BH4: Tetrahydrobiopterin
PTMs: Posttranslational modifications
PUFAs: Polyunsaturated fatty acids
NADPH: Nicotinamide adenine dinucleotide phosphate
GSH: Glutathione
SLC7A11: Solute carrier family 7 member 11
PDAC: Pancreatic ductal adenocarcinoma
ALOX12: Arachidonate (12S)-lipoxygenase
IFN*γ*: Interferon gamma
IRF1: Interferon regulatory factor 1
RTAs: Radical-trapping agents
4-CBA: 4-chlorobenzoic acid
PTS: Pyruvoyltetrahydropterin synthase
SPR: Sepiapterin reductase
NADP: Nicotinamide-adenine dinucleotide phosphate
GSSG: Oxidized GSH
IKE: Imidazole ketone erastin
PE: Piperazine erastin
FSP1: Ferroptosis suppressor protein 1
ROS: Reactive oxygen species
MEFs: Mouse embryonic fibroblasts
AMPK: AMP-activated protein kinase
I/R: Ischemia-reperfusion
ACC: Acetyl-CoA carboxylase
MAP1LC3B: Microtubule-associated protein 1 light chain 3
TLR4: Toll-like receptor 4
NF-*κ*B: Nuclear factor kappa-light-chain-enhancer of activated B cells
BMDMs: Bone marrow-derived macrophages
TNF-*α*: Tumor necrosis factor-*α*
IL-1*β*: Interleukin-1*β*
LPS: Lipopolysaccharide
IKK*β*: I*κ*B kinase *β*
eIF2*α*: Eukaryotic initiation factor 2*α*
TRIF: TIR domain-containing adapter molecule 1
IFN: Type I interferon
HIF: Hypoxia-inducible factor
Akt: Protein kinase B
GSK-3*β*: Glycogen synthase kinase 3*β*
Nrf2: NFE2-related factor 2
HSPB1: Heat shock protein beta-1
BAP1: BRCA1-associated protein 1
ATF4: Activating transcription factor 4
PRC1: Polycomb repressive complex 1
USP7: Ubiquitin-specific peptidase 7
OTUB1: OTU deubiquitinase ubiquitin aldehyde binding 1
AR: Androgen receptor
FBXL2: F-box and the leucine-rich repeat protein 2
HDAC: Histone deacetylase
HMGB1: High-mobility group box 1
DAMP: Damage-associated molecular pattern
HNF4A: Hepatocyte nuclear factor 4 alpha
HIC1: Hypermethylated in cancer 1
KDM3B: Lysine demethylase 3B
BRD4: Bromodomain-containing 4
EHMT2: Euchromatic histone lysine methyltransferase 2.
